# Short-Stay Hospitalizations for Patients with COVID-19: A Retrospective Cohort Study

**DOI:** 10.3390/jcm10091966

**Published:** 2021-05-03

**Authors:** Austin S. Kilaru, Kathleen Lee, Lindsay Grossman, Zachary Mankoff, Christopher K. Snider, Eric Bressman, Stefanie B. Porges, Keith C. Hemmert, Scott R. Greysen, David A. Asch, Mucio K. Delgado

**Affiliations:** 1 Center for Emergency Care Policy and Research, Department of Emergency Medicine, Perelman School of Medicine, University of Pennsylvania, Philadelphia, PA 19104, USA; kathleen.lee@pennmedicine.upenn.edu (K.L.); stefanie.porges@pennmedicine.upenn.edu (S.B.P.); keith.hemmert@pennmedicine.edu (K.C.H.); mucio.delgado@pennmedicine.upenn.edu (M.K.D.); 2 National Clinical Scholars Program, Corporal Michael J. Crescenz Philadelphia VA Medical Center, Philadelphia, PA 19104, USA; eric.bressman@pennmedicine.edu; 3 Perelman School of Medicine, University of Pennsylvania, Philadelphia, PA 19104, USA; lindsay.grossman@pennmedicine.upenn.edu (L.G.); zachary.mankoff@pennmedicine.upenn.edu (Z.M.); 4 Penn Medicine Center for Health Care Innovation, Philadelphia, PA 19104, USA; christopher.snider@pennmedicine.upenn.edu (C.K.S.); asch@wharton.upenn.edu (D.A.A.); 5 Penn Medicine Center for Evidence-Based Practice, Section of Hospital Medicine, Division of General Internal Medicine, Perelman School of Medicine, University of Pennsylvania, Philadelphia, PA 19104, USA; ryan.greysen@pennmedicine.upenn.edu

**Keywords:** hospital operations, hospital capacity, COVID-19, hospital readmissions

## Abstract

Objective: Patients requiring hospital care for COVID-19 may be stable for discharge soon after admission. This study sought to describe patient characteristics associated with short-stay hospitalization for COVID-19. Methods: We performed a retrospective cohort study of patients with COVID-19 admitted to five United States hospitals from March to December 2020. We used multivariable logistic regression to identify patient characteristics associated with short hospital length-of-stay. Results: Of 3103 patients, 648 (20.9%) were hospitalized for less than 48 h. These patients were significantly less likely to have an age greater than 60, diabetes, chronic kidney disease; emergency department vital sign abnormalities, or abnormal initial diagnostic testing. For patients with no significant risk factors, the adjusted probability of short-stay hospitalization was 62.4% (95% CI 58.9–69.6). Conclusion: Identification of candidates for early hospital discharge may allow hospitals to streamline throughput using protocols that optimize the efficiency of hospital care and coordinate post-discharge monitoring.

## 1. Introduction

Although significant attention has been paid to risk factors associated with mortality and severe illness due to COVID-19, most infected patients never need hospitalization, and many who do require only short hospital stays [1,2,3]. Indications for brief hospitalization in this group include transient vital sign abnormalities, symptom management, treatment of co-morbid illness, intravenous therapies, and potential risk of deterioration [4,5].

During the pandemic, hospitals have had to expand their capacity and streamline patient throughput to manage high patient volumes and preserve access to care unrelated to COVID-19 [6]. Short hospital stays, defined as hospitalization lasting less than 48 h, pose an operational challenge. The rapid turnover of patients compresses the time-intensive processes of admission and discharge, including arranging safe transitions to post-acute care. Inefficient patient progression may unnecessarily prolong length-of-stay and divert resources. However, patients who are discharged prematurely may be at risk for complications or readmission.

Anticipation of admitted patients with COVID-19 who are candidates for early discharge may allow hospitals to develop protocols to further reduce length-of-stay, involving strategies such as early discharge planning, protocolized delivery of therapeutic medications, and the use of dedicated observation units and personnel [7]. Improved identification of short-stay patients also enables continuous quality improvement. However, there is little evidence on patient factors associated with short hospital stays for moderate illness due to COVID-19 [4].

The goal of this study was to describe patient characteristics associated with short-stay hospitalization for COVID-19 among patients admitted from the emergency department to non-critical care units, to aid the development of efficient hospital care pathways for COVID-19. In a secondary analysis, we determined whether short-stay hospitalizations are associated with rehospitalization within seven days of discharge.

## 2. Materials and Methods

We conducted a retrospective cohort study of patients with COVID-19 requiring hospitalization at five hospitals within a multi-hospital health system in the United States. This study followed the Strengthening the Reporting of Observational Studies in Epidemiology guidelines [8]. 

We used electronic health record data to identify inpatient admission or observation encounters originating in the emergency department (ED) between March 1— December 31 2020. We apply the term ‘hospitalization’ to signify hospital encounters regardless of inpatient or observation status. Patients were included in the cohort if they tested positive for COVID-19 during the ED encounter, or, for patients who were tested as outpatients, up to seven days prior. In this system, hospital admission decisions were made at the discretion of the attending emergency physician based on evolving experience and evidence on the management of COVID-19 during this period. We excluded elective or surgical admissions and patients younger than 18 years. We excluded patients admitted from the ED directly to a critical care unit or service. We excluded patients who expired or were referred to hospice within 48 h of hospitalization.

The binary primary outcome was whether or not the hospital length-of-stay was 48 h or less. We defined the start of hospitalization as the time when the ED clinician presented the case to the inpatient medical team, representing the time point when the hospital team assumes responsibility for patient management. Patients may have physically remained in the ED for some period after this timepoint. We defined the end of hospitalization as the time of the hospital discharge order. In a secondary analysis, the outcome was whether patients were re-hospitalized within 7 days, among patients who did not expire or were discharged to hospice during the index hospitalization.

We selected covariates available in the electronic health record prior to the analysis to reflect patient characteristics that were common and known at the time of ED disposition. Covariates were selected a priori, and all were entered into the analysis. Variables were selected by the study team for several factors, including (1) previous evidence demonstrating an association with the severity of illness due to COVID-19, (2) availability of data for most patients hospitalized with COVID-19, and (3) a plausible explanatory association with the outcome of short-stay hospitalization. We included demographics, including age, sex, and self-reported race/ethnicity. We also included patient co-morbidities that we deemed might affect hospital length-of-stay: hypertension, diabetes, chronic kidney disease, chronic obstructive pulmonary disease (COPD) or asthma, and venous thromboembolism. These co-morbidities were included as binary variables according to their presence or absence as recorded at the time of the hospital admission. We also included physical signs (hypoxia, tachycardia, tachypnea, or fever in the ED) and results of common diagnostic testing (white blood cell count, serum lactate, blood urea nitrogen, and presence of pneumonia on chest radiograph); the parameters for normal and abnormal values are listed in the Supplementary Material. To account for confounding due to variation in the management of COVID-19 as the pandemic evolved as well as variation across hospital sites, the models were adjusted for month and hospital site.

We summarized unadjusted outcomes using descriptive statistics. For the primary adjusted analysis, we used multivariable logistic regression to compare the characteristics of patients who had a short-stay hospitalization to those who did not (binary outcome). In addition to reporting the adjusted odds ratios (AORs), we used marginal effects to report the adjusted probability of short-stay hospitalization with 95% confidence intervals (CIs) to more clearly quantify the effects of risk factors for the primary outcome [9]. For the secondary analysis, we used multivariable logistic regression to determine whether short hospital length-of-stay was associated with rehospitalization within 7 days of hospital discharge, and adjusted for patient demographics and co-morbidities. All analyses were conducted using Stata, version 16.1 (StataCorp LLC, College Station, TX). A *p*-value of less than 0.05 was considered statistically significant.

## 3. Results

Of the 3103 patients included in the study, 648 (20.9%) had a short-stay hospitalization. Patient characteristics are shown in Table 1. In the adjusted analysis, compared to patients aged 18–59, patients age > 60 years had significantly decreased odds of short-stay hospitalization (AOR 0.58 95%CI 0.46–0.72) (Table 2). Patients with a history of diabetes (AOR 0.71, 95%CI 0.55–0.91) or chronic kidney disease (AOR 0.50, 95%CI 0.34–0.72) also had significantly decreased odds of short-stay hospitalization, as did patients with abnormal vital signs in the ED, including hypoxia (AOR 0.57, 95%CI 0.46–0.71), tachycardia (AOR 0.47, 95%CI 0.39–0.58), tachypnea, (AOR 0.80, 95%CI 0.65–0.98), and fever (AOR 0.69, 95%CI 0.55–0.87). Finally, patients with leukocytosis (AOR 0.64, 95%CI 0.42–0.96), elevated blood urea nitrogen (AOR 0.52, 95%CI 0.40–0.67), and chest radiograph with clear or indeterminate evidence of pneumonia (AOR 0.54, 95%CI 0.44–0.66) had significantly decreased odds of short-stay hospitalization.

In this cohort, 191 patients had no risk factors found to be significantly associated with prolonged hospitalization. For those patients, the adjusted probability of short-stay hospitalization was 62.4% (95%CI 58.9–69.6). For the 238 patients with normal ED vital signs and diagnostic testing, the adjusted probability of short-stay hospitalization was 51.7% (95%CI 46.9–56.7). The adjusted probability of short-stay hospitalization for the 913 patients with an age of < 60, no history of diabetes, and no history of chronic kidney disease was 28.1% (95%CI 25.2–30.9).

In the secondary analysis, there were 2792 patients who did not expire or were discharged to hospice during the index hospitalization. Of 648 patients with index short-stay hospitalization, 48 (7.4%) required rehospitalization within 7 days. Of 2148 patients with index hospitalization greater than 48 h, 42 (2.0%) required rehospitalization. Short-stay hospitalization had significantly increased odds of rehospitalization within 7 days (AOR 4.9, 95%CI 3.1–7.7). Additional patient characteristics associated with 7-day rehospitalization are provided in Supplemental Table S2.

## 4. Discussion

In this study, 1 in 5 patients with COVID-19 admitted from the ED to medical wards were discharged within 48 h. Several patient characteristics were independently associated with a greater likelihood of short-stay hospitalization. Furthermore, we found that patients younger than 60 with normal ED vital signs and diagnostic testing had greater than a 50% probability of discharge within 48 h. These patient groups may be appropriate candidates for targeted short-stay pathways that seek to further streamline throughput and reduce length-of-stay. The presence of such pathways might lead to even further reductions in hospital length-of-stay, both for patients who may meet discharge criteria before 48 h but who are not discharged by that time as well as patients who are discharged within 48 h but may have been ready to depart even sooner.

During the pandemic, hospitals cohorted patients with COVID-19 to minimize the spread of infection [10]. Further cohorting of patients with anticipated short length-of-stay may allow hospitals to develop, test, and refine new protocols that ensure timely yet appropriate discharge. These protocols may include early discharge planning, frequent patient reevaluation, and focused delivery of disease-specific interventions, such as novel intravenous therapies and early specialist consultation. The cohorting of short-stay patients may also allow other clinicians to devote greater attention to patients with more severe illness rather than coordinate discharge plans.

For other disease conditions, the use of dedicated observation units for short hospital encounters is widespread in the United States, and observation protocols are widely recognized to create efficient delivery of hospital-level services [11]. Observation criteria for COVID-19 have not been clearly defined but are needed—increased hospital efficiency continues to be needed to maintain hospital capacity for both COVID and non-COVID patients, during current and future resurgences.

This study also found that 7% of patients discharged within 48 h were readmitted compared to 2% of patients with longer index hospitalization. Prior evidence suggests a similar risk of readmission during the immediate period following hospital discharge, although studies examining a longer time period have shown higher rates of readmission [12,13,14]. Indications for readmission may include the progression of acute COVID-19 illness, sequelae of the initial hospitalization, such as deconditioning or hypoxia, potential long-term complications, and consequences of the pandemic for the healthcare system, including limited access to outpatient services [13]. It may be expected that patients discharged after short-stay hospitalizations have a higher likelihood of readmission, given that the unpredictable course of COVID-19 may pose a risk of deterioration to patients initially deemed stable for discharge. However, this increased risk may be warranted and, in the absence of ongoing indications for hospitalization, patients may need to be discharged to maintain hospital capacity for COVID and non-COVID patients alike [15]. Close monitoring after discharge may also mitigate the risk of readmission. Given the challenges of outpatient evaluation of patients with active infection, telemedicine services may be well-suited for this purpose. Home pulse oximetry and automated text message systems have been proposed as mechanisms for following patients after hospital discharge [16,17].

This study has several limitations. First, approaches to the management of COVID-19 varied significantly over the course of the study period—first, due to the uncertainty of patient trajectories and, later, due to the introduction of intravenous therapies [18]. Second, protocols varied between hospitals and practices varied among clinicians. Therefore, the patients included in this study did not have consistent criteria for hospitalization. Similarly, patients were discharged from the hospital without strict criteria or protocols. It is likely that some patients discharged after 48 h may have been eligible for discharge within the short-stay window as well as the converse. Furthermore, we did not adjust for hospital factors, like crowding, which might affect length-of-stay. To partially mitigate these sets of confounders, we adjusted for the month and hospital site. In addition, we did not identify rehospitalizations at facilities external to this health system. Finally, this analysis was intended as descriptive rather than for the purposes of prediction, limiting its generalizability.

## 5. Conclusions

In summary, patients with moderate–severe illness due to COVID-19 may only require brief hospitalization. Patient characteristics and initial ED evaluation may help hospitals distinguish these patients and develop care pathways that optimize efficient care. Given the increased risk of rehospitalization after brief hospitalization, patients may benefit from close monitoring after discharge.

## Figures and Tables

**Table 1:** Patient characteristics for non-critical care COVID-19 hospitalizations by length-of-stay (*n* = 3103).

		Hospitalization < 48 h, *n* (%) *n* = 648 (20.9%)	Hospitalization > 48 h, *n* (%) *n* = 2455 (79.1%)	*p*
**Patient demographics**				
Age				<0.001
	18–59 years	374 (57.7)	882 (35.9)	
	>60 years	274 (42.3)	1573 (64.1)	
Sex				0.30
	Female	318 (49.1)	1261 (51.4)	
	Male	330 (50.9)	1194 (48.6)	
Race/Ethnicity				0.08
	Non-Hispanic White	231 (35.7)	987 (40.2)	
	Non-Hispanic Black	297 (45.8)	1035 (42.2)	
	Hispanic/Latino	62 (9.6)	225 (9.2)	
	Asian	24 (3.7)	116 (4.7)	
	Other	34 (5.3)	92 (3.8)	
Insurance				<0.001
	Commercial/self-pay	275 (42.4)	688 (28.0)	
	Medicare	232 (35.8)	1400 (57.0)	
	Medicaid	141 (21.8)	367 (15.0)	
**History of co-morbid illness**				
Hypertension		258 (39.8)	1208 (49.2)	<0.001
Diabetes		131 (20.2)	744 (30.3)	<0.001
Chronic kidney disease		47 (7.3)	437 (17.8)	<0.001
COPD/Asthma		82 (12.7)	398 (16.2)	0.03
Venous thromboembolism		44 (6.8)	225 (9.2)	0.06
**Emergency Department Vital Signs**				
Hypoxia (minimum O2 saturation < 94%)		249 (38.4)	1528 (62.2)	<0.001
Tachycardia (maximum heart rate > 100 beats per min)		316 (48.8)	1665 (67.8)	<0.001
Tachypnea (maximum respiratory rate > 20 breaths per min)		302 (46.6)	1564 (63.7)	<0.001
Fever (maximum temperature > 100.4 degrees Fahrenheit)		130 (20.1)	835 (34.0)	<0.001
**Emergency Department Vital Signs and Test Results**				
Leukocytosis		35 (5.4)	220 (9.0)	0.003
Elevated blood urea nitrogen		135 (20.8)	1069 (43.5)	<0.001
Elevated serum lactate		71 (11.0)	418 (17.0)	<0.001
Chest radiograph				<0.001
	No evidence of pneumonia	388 (59.9)	830 (33.8)	
	Evidence of pneumonia or indeterminate	260 (40.1)	1625 (66.2)	

Leukocytosis: white blood cell count greater than 12,000 cells per mcL; elevated blood urea nitrogen: greater than 20 mg/dL; elevated serum lactate: lactate greater than 2 mmol/L. SD: standard deviation; COPD: chronic obstructive pulmonary disease.

**Table 2:** Adjusted odds ratios and adjusted probability of short-stay hospitalization for COVID-19 for patient characteristics.

		Adjusted Odds Ratio (95% CI)	*p*	Adjusted Probability, % (95% CI)
**Age**	18–59 years	reference	--	25.1 (22.8–27.4)
	>60 years	0.58 (0.46–0.72)	<0.001	17.3 (15.5–19.1)
**Sex**	Male	reference	--	22.2 (20.3–24.2)
	Female	0.82 (0.68–1.00)	0.05	19.6 (17.8–21.4)
**Race/Ethnicity**	Non-Hispanic White	reference	--	19.8 (17.6–22.1)
	Non-Hispanic Black	1.16 (0.90–1.50)	0.24	21.9 (17.6–24.2)
	Hispanic/Latino	1.02 (0.71–1.46)	0.94	20.0 (15.7–24.3)
	Asian	0.92 (0.56–1.54)	0.76	18.8 (12.6–25.0)
	Other	1.34 (0.83–2.17)	0.23	24.0 (17.3–30.7)
**Hypertension**	No	reference	--	20.9 (19.0–22.8)
	Yes	1.00 (0.80–1.25)	0.76	20.9 (18.7–23.1)
**Diabetes**	No	reference	--	22.1 (20.5–23.7)
	Yes	0.71 (0.55–0.91)	0.006	17.5 (14.8–20.1)
**Chronic kidney disease**	No	reference	--	22.0 (20.5–23.5)
	Yes	0.50 (0.34–0.72)	< 0.001	13.4 (9.9–17.0)
**COPD/Asthma**	No	reference	--	21.3 (19.9–22.8)
	Yes	0.80 (0.60–1.07)	0.14	18.4 (15.1–21.8)
**Venous thromboembolism**	No	reference	--	21.3 (19.9–22.7)
	Yes	0.69 (0.47–1.01)	0.05	16.6 (12.4–20.8)
**Hypoxia in ED**	No	reference	--	25.0 (22.8–27.3)
	Yes	0.57 (0.46–0.71)	<0.001	17.0 (15.2–18.9)
**Tachycardia in ED**	No	reference	--	27.7 (25.2–30.1)
	Yes	0.47 (0.39–0.58)	<0.001	16.8 (15.2–18.4)
**Tachypnea in ED**	No	reference	--	22.5 (20.4–24.7)
	Yes	0.80 (0.65–0.98)	0.04	19.5 (17.6–21.3)
**Fever in ED**	No	reference	--	22.2 (20.6–23.8)
	Yes	0.69 (0.55–0.87)	0.002	17.3 (14.8–19.7)
**Leukocytosis**	No	reference	--	21.3 (19.9–22.7)
	Yes	0.64 (0.42–0.96)	0.03	15.7 (11.2–20.1)
**Elevated blood urea nitrogen**	No	reference	--	23.7 (21.9–25.5)
	Yes	0.52 (0.40–0.67)	< 0.001	15.0 (12.7–17.3)
**Elevated serum lactate**	No	reference	--	21.3 (19.9–22.7)
	Yes	0.79 (0.58–1.01)	0.13	18.1 (14.6–21.7)
**Chest radiograph**	Normal/not performed	reference	--	25.8 (23.5–28.1)
	Abnormal	0.54 (0.44–0.66)	<0.001	16.8 (15.1–18.6)

Model adjusted for month of presentation and hospital site within the health system, with results listed in the Supplementary Materials. Hypoxia: minimum oxygen saturation less than 94%; tachycardia: heart rate greater than 100 beats per minute; tachypnea: respiratory rate greater than 20 breaths per minute; fever: temperature greater than 100.4 degrees Fahrenheit; leukocytosis: white blood cell count greater than 12,000 cells per mcL; elevated BUN: greater than 20 mg/dL; elevated lactate: greater than 2 mmol/L; COPD: chronic obstructive pulmonary disease; ED: emergency department.

## Data Availability

The data presented in this study are available on request from the corresponding author. The data are not publicly available due to patient privacy and health system confidentiality.

## Supplementary Materials

The following are available online at https://www.mdpi.com/article/10.3390/jcm10091966/s1, Table S1. Covid-19 non-critical care hospitalizations by month and hospital site, with adjusted odds ratios (*n* = 3103); Table S2. Association of patient characteristics with rehospitalization within 7 days following index hospitalization for Covid-19 not resulting in death or discharge to hospice (*n* = 2792).
