# Supplementary material for: Short-Stay Hospitalizations for Patients with COVID-19: A Retrospective Cohort Study
Source: J Clin Med. 2021 May 3;10(9):1966. doi: 10.3390/jcm10091966 (PMC8125769; doi:10.3390/jcm10091966)
Supplement: Supplementary file 1 [file jcm-10-01966-s001.zip › jcm-1178292-supplementary.pdf]

Web Supplement

1. Parameters for vital signs and diagnostic testing
2. Month and hospital site results (unadjusted and adjusted)
3. Secondary outcome logistic regression model

Parameters for vital signs and diagnostic testing

Hypoxia, minimum ED pulse oximeter reading <94% regardless of supplemental oxygenation.

Tachycardia, maximum ED heart rate >100 beats per minute.

Tachypnea, maximum ED respirator rate >20 breaths per minute.

Fever, maximum ED recorded temperature greater than or equal to 100.4 degrees Fahrenheit.

Leukocytosis, white blood cell count greater than 12,000 cells per mcL.

Elevated blood urea nitrogen, greater than 20 mg/dL.

Elevated serum lactate, greater than 2 mmol/L.

Chest radiograph results were classified as 1) normal / not performed or 2) abnormal / indeterminate with regard to the presence of pneumonia (consolidation, opacities, infiltrates) by two independent coders based on the final interpretation of the attending radiologist. Normal radiographs were defined as radiographs with no active acute disease, or isolated findings not associated with Covid-19 pneumonia such as pneumothorax. Abnormal radiographs were defined as radiographs with clear evidence of Covid-19 pneumonia, such as multifocal opacities. Indeterminate radiographs were those for which the attending radiologist could not distinguish active illness from benign processes, such as atelectasis.

Table S1. Covid-19 non-critical care hospitalizations by month and hospital site, with adjusted odds ratios (*n* = 3103).

Covariate	Hospitalization < 48 hours, *n* (%) *n* = 648 (20.9%)	Hospitalization > 48 hours, *n* (%) *n* = 2455 (79.1%)	Adjusted Odds Ratio (95% CI)	*p*
**Month**				
March 2020	26 (4.0)	166 (6.8)	reference	--
April	106 (16.4)	589 (24.0)	0.95 (0.58–1.56)	0.84
May	85 (13.1)	306 (12.5)	1.22 (0.73–2.05)	0.45
June	43 (6.6)	110 (4.5)	1.36 (0.74–2.48)	0.32
July	31 (4.8)	88 (3.6)	1.13 (0.60–2.14)	0.71
August	28 (4.3)	55 (2.2)	1.51 (0.77–3.00)	0.23
September	18 (2.8)	47 (1.9)	1.23 (0.58–2.61)	0.58
October	34 (5.3)	117 (4.8)	1.14 (0.62–2.10)	0.67
November	99 (15.3)	420 (17.1)	1.12 (0.67–1.85)	0.67
December	178 (27.5)	557 (22.7)	1.51 (0.93–2.46)	0.09
Hospital				
A	147 (22.7)	432 (17.6)	reference	
B	158 (24.4)	554 (22.6)	0.99 (0.74–1.34)	0.95
C	107 (16.5)	338 (13.8)	0.90 (0.65–1.25)	0.53
D	124 (19.1)	487 (19.8)	0.99 (0.71–1.39)	0.98
E	112 (17.3)	644 (26.2)	0.51 (0.37–0.70)	0.001

Table S2. Association of patient characteristics with rehospitalization within 7 days following index hospitalization for Covid-19 not resulting in death or discharge to hospice (n = 2792).

		**Adjusted Odds Ratio (95% CI)**	***p***
**Index hospitalization length-of-stay**			
	**> 48 hours**	**reference**	
	**< 48 hours**	**4.88 (3.10–7.67)**	**<0.001**
**Age**	18–59 years	reference	--
	>60 years	1.98 (1.18–3.33)	0.009
**Sex**	Male	reference	--
	Female	0.60 (0.39–0.95)	0.03
**Race/Ethnicity**	Non-Hispanic White	reference	--
	Non-Hispanic Black	0.67 (0.38–1.21)	0.19
	Hispanic/Latino	1.13 (0.50–2.59)	0.77
	Asian	0.28 (0.04–2.11)	0.22
	Other	0.49 (0.11–2.14)	0.34
**Hypertension**	No	reference	--
	Yes	1.47 (0.88–2.45)	0.14
**Diabetes**	No	reference	--
	Yes	0.78 (0.46–1.32)	0.36
**Chronic kidney disease**	No	reference	--
	Yes	1.46 (0.80–2.69)	0.22
**COPD/asthma**	No	reference	--
	Yes	2.26 (1.35–3.79)	0.002
**Venous thromboembolism**	No	Reference	--
	Yes	0.43 (0.11–1.12)	0.08
**Month**			
	March 2020	reference	--
	April	0.83 (0.26–2.63)	0.75
	May	0.63 (0.18–2.19)	0.46
	June	0.88 (0.22–3.49)	0.85
	July	0.68 (0.14–3.29)	0.64
	August	0.90 (0.19–4.32)	0.89
	September	2.14 (0.48–9.50)	0.32
	October	1.15 (0.31–4.28)	0.84
	November	1.20 (0.39–3.70)	0.74
	December 2020	0.71 (0.23–2.19)	0.55
**Hospital site**			
	A	reference	--
	B	1.34 (0.66–2.72)	0.42
	C	1.00 (0.45–2.21)	0.99
	D	1.50 (0.72–3.10)	0.27
	E	0.57 (0.23–1.40)	0.22
